# Thymopentin-Mediated Inhibition of Cancer Stem Cell Stemness Enhances the Cytotoxic Effect of Oxaliplatin on Colon Cancer Cells

**DOI:** 10.3389/fphar.2022.779715

**Published:** 2022-02-15

**Authors:** Peng-Cheng Yu, Di Liu, Zeng-Xiang Han, Fang Liang, Cui-Yun Hao, Yun-Tao Lei, Chang-Run Guo, Wen-Hui Wang, Xing-Hua Li, Xiao-Na Yang, Chang-Zhu Li, Ye Yu, Ying-Zhe Fan

**Affiliations:** ^1^ Interventional Cancer Institute of Chinese Integrative Medicine, Putuo Hospital, Shanghai University of Traditional Chinese Medicine, Shanghai, China; ^2^ School of Basic Medicine and Clinical Pharmacy, China Pharmaceutical University, Nanjing, China; ^3^ Three Departments of Oncology, Weifang Traditional Chinese Medicine Hospital, Weifang, China; ^4^ State Key Laboratory of Utilization of Woody Oil Resource, Hunan Academy of Forestry, Changsha, China

**Keywords:** thymopentin (TP5), colon cancer cells, oxaliplatin, AChRs, cancer stem cell (CSC)

## Abstract

Thymopentin (TP5) is an immunomodulatory pentapeptide that has been widely used in malignancy patients with immunodeficiency due to radiotherapy and chemotherapy. Here, we propose that TP5 directly inhibits the stemness of colon cancer cells HCT116 and therefore enhances the cytotoxicity of oxaliplatin (OXA) in HCT116 cells. In the absence of serum, TP5 was able to induce cancer stemness reduction in cultured HCT116 cells and significantly reduced stemness-related signals, such as the expression of surface molecular markers (CD133, CD44 and CD24) and stemness-related genes (ALDH1, SOX2, Oct-4 and Nanog), and resulted in altered Wnt/β-catenin signaling. Acetylcholine receptors (AchRs) are implicated in this process. OXA is a common chemotherapeutic agent with therapeutic effects in various cancers. Although TP5 had no direct effect on the proliferation of HCT116, this pentapeptide significantly increased the sensitivity of HCT116 to OXA, where the effect of TP5 on the stemness of colon cancer cells through stimulation of AchRs may contribute to this process. Our results provide a promising strategy for increasing the sensitivity of colon cancer cells to chemotherapeutic agents by incorporating immunomodulatory peptides.

## Introduction

Colorectal cancer (CRC) is one of the common malignant tumors of the gastrointestinal system, and the current 5-year survival rate of colorectal cancer is 66% worldwide, but the 5-year survival rate of patients with advanced disease is only 13% (Miller et al., 2019). Currently, the main treatments for colorectal cancer include surgical resection, radiotherapy, and immune anti-cancer therapy (Ciombor et al., 2015). Numerous studies have shown that cancer stem cells (CSCs) are the core factor leading to postoperative recurrence, radiotherapy insensitivity, and immunotherapy resistance in tumor patients (Regan et al., 2017; Tosoni et al., 2017; Luo et al., 2018). Cancer stem cells are a very small subpopulation of tumor cells with unlimited self-renewal, multidirectional differentiation potential and high malignancy, which have been shown to be present in many types of tumors including colon cancer, and they confer tumor metastasis, chemoresistance and sustained adaptation to the microenvironment (Kreso and Dick, 2014; Fearon and Carethers, 2015).

During tumor treatment, cancer stem cells exhibit a high degree of insensitivity to chemotherapy and radiotherapy (Zhao, 2016). It has been found that colorectal cancer stem cells are intrinsically resistant to first-line chemotherapeutic agents for rectal cancer, such as 5-fluorouracil and oxaliplatin (OXA) (Hammond et al., 2016). In addition, cancer stem cells are theoretically mostly in a quiescent state, while oncologic therapies such as radiotherapy and chemotherapy target proliferating tumor cells (Phi et al., 2018). Therefore, in many colorectal cancer patients, although good therapeutic results can be achieved at the beginning of drug administration, an avalanche of multidrug resistance soon occurs and tumors progress rapidly (Hervieu et al., 2021). The mechanism of this phenomenon may be due to the fact that chemotherapeutic drugs do not act on cancer stem cells when killing proliferating tumor cells (Van der Jeught et al., 2018). This screening pressure creates a competitive advantage for cancer stem cells, thus allowing cancer stem cells and their derivatives to become the main body of advanced tumors, exacerbating tumor resistance, progression and metastasis (Ayob and Ramasamy, 2018). On the other hand, surgical resection of tumors or radiotherapy cannot guarantee the removal of all cancer stem cells, and a very small number of cancer stem cells can become tumorigenic cells. Therefore, cancer stem cells are an important cause of drug resistance in tumor treatment and of recurrence and metastasis after treatment (Zhu et al., 2009; Lytle et al., 2019; Clara et al., 2020). Colon cancer stem cells can express specific surface markers such as CD24, CD44 and CD133, which are highly resistant to radiotherapy and chemotherapy (Du et al., 2008). ALDH1, OCT4, SOX2 and Nanog are all markers of the stemness state of cancer stem cells and are important in maintaining the nature and drug resistance of cancer stem cells (Chambers et al., 2003; Tirosh et al., 2016; Anorma et al., 2018; Bocci et al., 2019).

TP5 is a synthetic pentapeptide derived from the active fragment of thymosin (49 amino acids), which exhibits very strong immunomodulatory activity in many animal models and *in vitro* assays of human cells (Patruno et al., 2012; Zhang et al., 2019). Numerous *in vivo* studies have shown that TP5 can be effective in the treatment of various diseases such as primary and secondary immunodeficiency, autonomic immunodeficiency, and severe infections (Fabrizi et al., 2006; Csaba, 2016). The combined application of chemotherapy and immunotherapy is a very effective modality in the treatment of tumors. TP5 can be used as a good immune adjuvant in the treatment of tumors, enhancing the immune system and reducing the side effects of chemotherapy and radiotherapy (Singh et al., 1998; Bodeya et al., 2000). In addition to the immune system, TP5 can also act directly on tumor cell-related signaling pathways (Li et al., 2010). Our previous study found that the immunomodulatory peptides thymosin and TP5 could inhibit the proliferation of leukemia cells and induce their differentiation into granulocytes (Fan et al., 2006a; Fan et al., 2006b).

In the present study, we found that TP5 can directly inhibit cultured colon cancer stem cells in medium without serum, and reduce the expression of cancer stem cell markers: CD44, CD24 and CD133, and stem-cell related genes: ALDH2, SOX2, OCT4 and Nanog. Additionally, although TP5 could not inhibit the proliferation of colon cancer cells directly, this peptide could promote the anti-proliferative effect of chemotherapeutic drug OXA on colon cancer cells HCT116, and acetylcholine receptors (AchRs) are implicated in this process.

## Results

### TP5 Inhibits the Formation of HCT116 Cancer Stem Cell Spheroids

**FIGURE 1:**
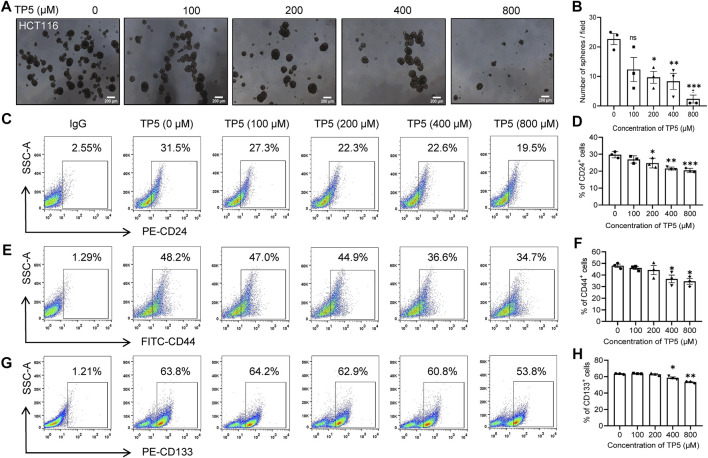
TP5 inhibits the stemness of cancer stem cells in HCT116 cells. **(A, B)** Representative photomicrographs of sphere formation experiments **(A)** and statistical analysis of the number of spheres with diameter ≥140 μm in visual field **(B)**. HCT116 cells were incubated with different treatments of TP5 (0, 100, 200, 400, and 800 μM) and grown in serum-free medium for 6 days. Scale bar = 200 μm. **(C–H)** Flow cytometer analysis of the expression of CD24, CD44 or CD133 **(C, E, G)** and pooled data of cell surface markers CD24 **(D)**, CD44 **(F)** and CD133 **(H)** (*n* = 3 independent experiments). All data are expressed as mean ± SD; ^*^ *p* < 0.05, ^**^ *p* < 0.01 and ^***^ *p* < 0.001 vs. control, one-way ANOVA with Dunnett’s post-hoc test, **(A)**, [F (4, 10) = 8.265, *p* = 0.0033], **(B)**, [F (4, 10) = 11.76, *p* = 0.0008] [F (4, 10) = 5.039 *p* = 0.0174], [F (4, 10) = 34.00, *p* < 0.0001].

Cancer stem cells have a very typical growth pattern in that they can be grown in suspension in serum-free medium with the addition of growth factors, eventually forming cancer stem cell spheroids with a three-dimensional structure. The ability to form spheroids is an important way to identify cancer stem cells *in vitro* (Masuda et al., 2016). Therefore, we investigated the effect of TP5 on spheroid formation of HCT116 cancer stem cells. The number of cancer stem cell spheroids was significantly less in HCT116 cells treated with TP5 than in the control group, and the spheroid formation rate was significantly decreased in a concentration-dependent manner (Figures 1A,B), indicating that TP5 reduced the stemness of HCT116 cancer stem cells and inhibited the self-renewal and proliferation ability of cancer stem cells.

### TP5 Reduces the Expression of Cancer Stem Cell Markers in HCT116 Cells

To further verify the effect of TP5 on colon cancer stem cells, we used flow cytometry to detect the expression of cancer stem cell markers CD24, CD44 and CD133, which are surface markers that can be specifically expressed by colon cancer stem cells (Jaggupilli and Elkord, 2012; Sahlberg et al., 2014). In the concentration range of 100–800 μM, TP5 could concentration-dependently decrease the expression of CD24, CD44 and CD133 in HCT116 cells (Figures 1C–H). The percentage of CD24-positive cells decreased from 29.7 ± 1.9% to 26.9 ± 2.1%, 24.7 ± 2.9%, 21.5 ± 0.9% and 20.4 ± 1.2%, after 48 h of action of 100, 200, 400, and 800 μM of TP5, respectively (Figures 1C,D). The percentage of CD44-positive cells decreased from 48.0 ± 2.4% to 46.0 ± 2.1%, 44.2 ± 6.6%, 36.6 ± 5.8%, and 34.5 ± 4.3%, respectively (Figures 1E,F). The percentage of CD133 positive cells decreased from 63.4 ± 0.5% to 63.9 ± 0.5%, 63.2 ± 1.5%, 58.5 ± 1.7%, and 53.4 ± 1.0%, respectively (Figures 1G,H).

### TP5 Inhibits LoVo Cancer Stem Cell Spheroid Formation and Reduces the Expression of Cancer Stem Cell Markers

**FIGURE 2:**
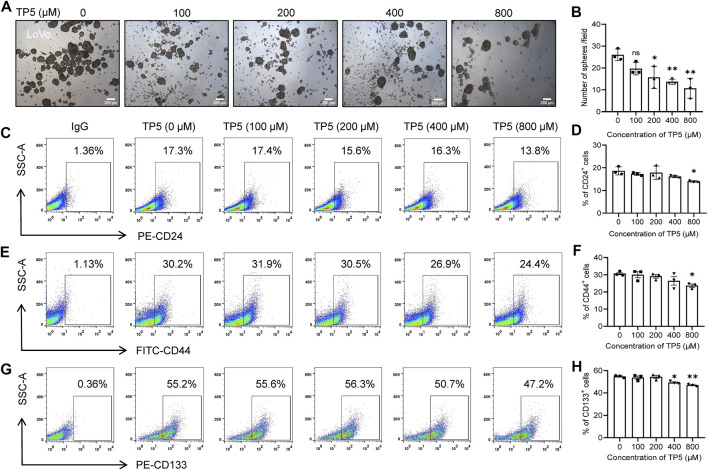
TP5 inhibits the stemness of cancer stem cells in LoVo cells. **(A, B)** Representative photomicrographs of sphere formation experiments **(A)** and statistical analysis of the number of spheres with diameter ≥140 μm in visual field **(B)**. LoVo cells were incubated with different treatments of TP5 (0, 100, 200, 400, and 800 μM) and grown in serum-free medium for 6 days. Scale bar = 200 μm. **(C–H)** Flow cytometer analysis of the expression of CD24, CD44 or CD133 **(C, E, G)** and pooled data of cell surface markers CD24 **(D)**, CD44 **(F)** and CD133 **(H)** (*n* = 3 independent experiments). All data are expressed as mean ± SD; ^*^ *p* < 0.05, ^**^ *p* < 0.01 versus control, one-way ANOVA with Dunnett’s post-hoc test, **(B)**, [F (4, 10) = 8.484, *p* = 0.0030], **(D)**, [F (4, 10) = 3.795, *p* = 0.0397], **(F)**, [F (4, 10) = 3.397 *p* = 0.0531], **(H)**, [F (4, 10) = 11.09, *p* = 0.0011].

We also investigated the effect of TP5 on cancer stem cell spheroid formation of another cell line, LoVo. The number of cancer stem cell spheroids was significantly less in LoVo cells treated with TP5 than in the control group, and the rate of spheroid formation was also significantly decreased (Figures 2A,B), indicating that TP5 also reduced the stemness of LoVo cancer stem cells. At a concentration of 800 μM, TP5 reduced the expression of CD24, CD44 and CD133 in HCT116 cells (Figures 1C–H). The percentage of CD24-positive cells decreased from 18.7 ± 1.8% to 14.0 ± 0.4% after 24 h of TP5 at 800 μM (Figures 2C,D). The percentage of CD44-positive cells decreased from 30.7 ± 1.3% to 23.7 ± 1.8%, respectively (Figures 2E,F). After 24 h of TP5 at 400 and 800 μM, the percentage of CD133 positive cells decreased from 54.8 ± 1.1% to 49.3 ± 1.2% and 47.2 ± 1.1%, respectively (Figures 2G,H). These results suggest that TP5 inhibition of cancer stem cells is not limited to HCT116 cells.

### TP5 Reduces the Expression of Cancer Stem Cell-Related Genes in HCT116 Cells

Although TP5 inhibits both HCT116 and LoVo cancer stem cells, it has a slightly stronger ability to inhibit HCT116 stem cell formation, we therefore focused on HCT116 as an example and continued to investigate the mechanism of TP5 inhibition of cancer stem cell formation and its application.

**FIGURE 3:**
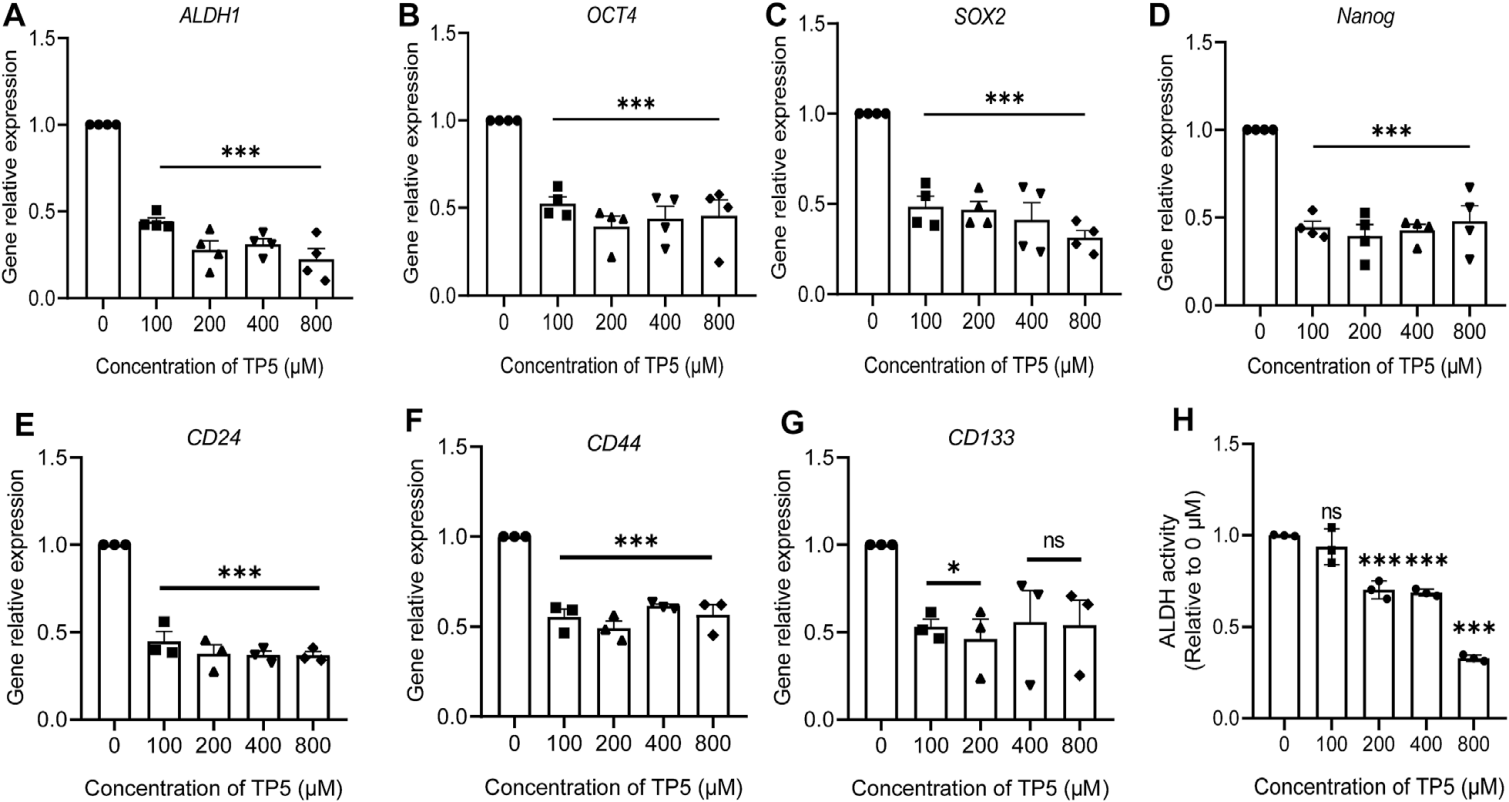
TP5 reduced the expression of cancer stem cell-related genes in HCT116 cells. **(A–G)** RT-qPCR analysis of relative mRNA expression of CSC-associated genes ALDH1 **(A)**, SOX-2 **(B)**, Oct-4 **(C)**, Nanog **(D)**, CD24 **(E), CD44 (F**) and CD133 **(G)** in HCT116 cells. **(H)** ALDH activity before and after treatment with different concentrations of TP5 (*n* = 3–4 independent experiments). All data are expressed as mean ± SD; ^*^ *p* < 0.05 and ^***^ *p* < 0.001 vs. control, one-way ANOVA with Dunnett’s post hoc test, **(A)**, F (4, 15) = 63.02, *p* < 0.0001, **(B)**, F (4, 15) = 17.52, *p* < 0.0001, **(C)**, F (4, 15) = 22.74, *p* < 0.0001, **(D)**, F (4, 15) = 22.83, *p* < 0.0001, **(E)**, F (4, 10) = 3.392, *p* = 0.0533, **(F)**, F (4, 10) = 53.61, *p* < 0.0001, **(G)**, F (4, 10) = 29.41, *p* < 0.0001, **(H)**, (F (4, 10) = 83.55, *p* < 0.0001; ns, not significant.

ALDH1, OCT4, SOX2 and Nanog are all stemness markers of cancer stem cells and have important roles in maintaining the nature of cancer stem cells and drug resistance (Anorma et al., 2018). We used Real-time PCR to detect the expression of cancer stem cell-related genes ALDH1, SOX-2, OCT-4, and Nanog. The expressions of ALDH1, SOX-2, OCT-4 and Nanog were significantly decreased after 100–800 μM TP5 were applied to HCT116 cells for 48 h (Figures 3A–D). It is suggested that TP5 may inhibit the self-renewal and tumorigenesis of colon cancer stem cells by regulating the important stemness genes ALDH1, SOX-2, OCT-4, and Nanog. Additionally, 100–800 μM TP5 also could significantly decrease the expression of CD24, CD44 and CD133 genes in HCT116 cells (Figures 3E–G). We also examined ALDH enzyme activity as a marker of cancer stem cell stemness (Jiang et al., 2019). TP5 concentration-dependently inhibited ALDH activity in HCT116 cells (after 48 h of TP5 action at 100, 200, 400, and 800 μM, ALDH activity decreased to 0.94 ± 0.10, 0.70 ± 0.05, 0.69 ± 0.02 and 0.33 ± 0.02; Figure 3H).

### Effect of TP5 on Wnt/β-Catenin Signaling Pathway

**FIGURE 4:**
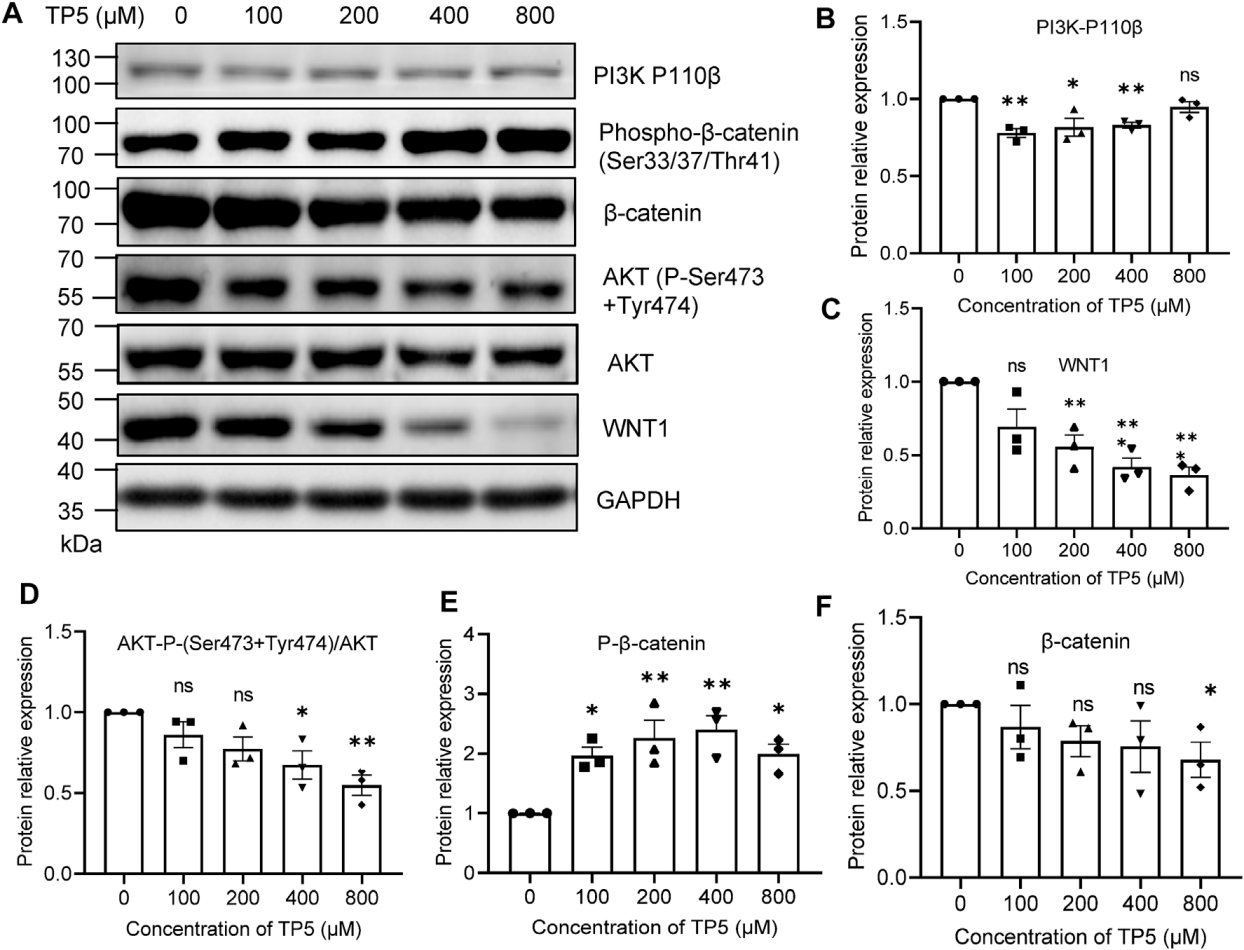
Effect of TP5 on Wnt/β-catenin signaling pathway. **(A–F)** Typical Western blot analysis **(A)** and pooled data of protein expression levels of PI3K-P110 β **(B)**, WNT1**(C)**, P-AKT**(D)**, P-β-catenin **(E)** and β-catenin **(F)**. Histograms are expressed as induction rates in triplicate for the GAPDH control. All data are expressed as mean ± SD; ^*^ *p* < 0.05, ^**^ *p* < 0.01 and ^***^ *p* < 0.001, one-way ANOVA with Dunnett’s post hoc test, **(B)**, F (4, 10) = 7.486, *p* = 0.0047, **(C)**, F (4, 10) = 11.47, *p* = 0.0009, **(D)**, F (4, 10) = 6.387, *p* = 0.0081, **(E)**, F (4, 10) = 7.421, *p* = 0.0048, **(F)**, F (4, 10) = 1.342, *p* = 0.3204; ns, not significant.

The Wnt signaling pathway is a key pathway for cancer stem cell development, and the sustained activation of the Wnt signaling pathway leads to the generation of cancer stem cells, which is an important factor for tumor resistance to conventional chemotherapy. Therefore, blocking the Wnt signaling pathway may be the key to removing cancer stem cells for the treatment of colon cancer (Polakis, 2007). β-catenin is a key downstream effector molecule in the Wnt signaling pathway (Liu et al., 2002). After the action of TP5 at 100–800 μM (Figure 4), HCT116 cells exhibited decreased PI3K (110 β) expression (100–400 μM effective), decreased AKT (P-Ser473 + Tyr474) phosphorylation levels (400–800 μM effective), and decreased WNT1 expression (200–800 μM effective); phosphorylated β-catenin (Ser33/37/Thr41) was increased (100–800 μM effective), leading to β-catenin degradation and expression decreased (800 μM effective) (the slightly different sensitivity of these indicators to TP5 may be due to some complex association of these factors), suggesting that TP5 inhibited colon cancer stem cells by affecting the wnt/β-catenin signaling pathway, which in turn inhibited colon cancer stem cells.

### TP5 Reduces Intracellular Calcium Concentration in HCT116 Cells

**FIGURE 5:**
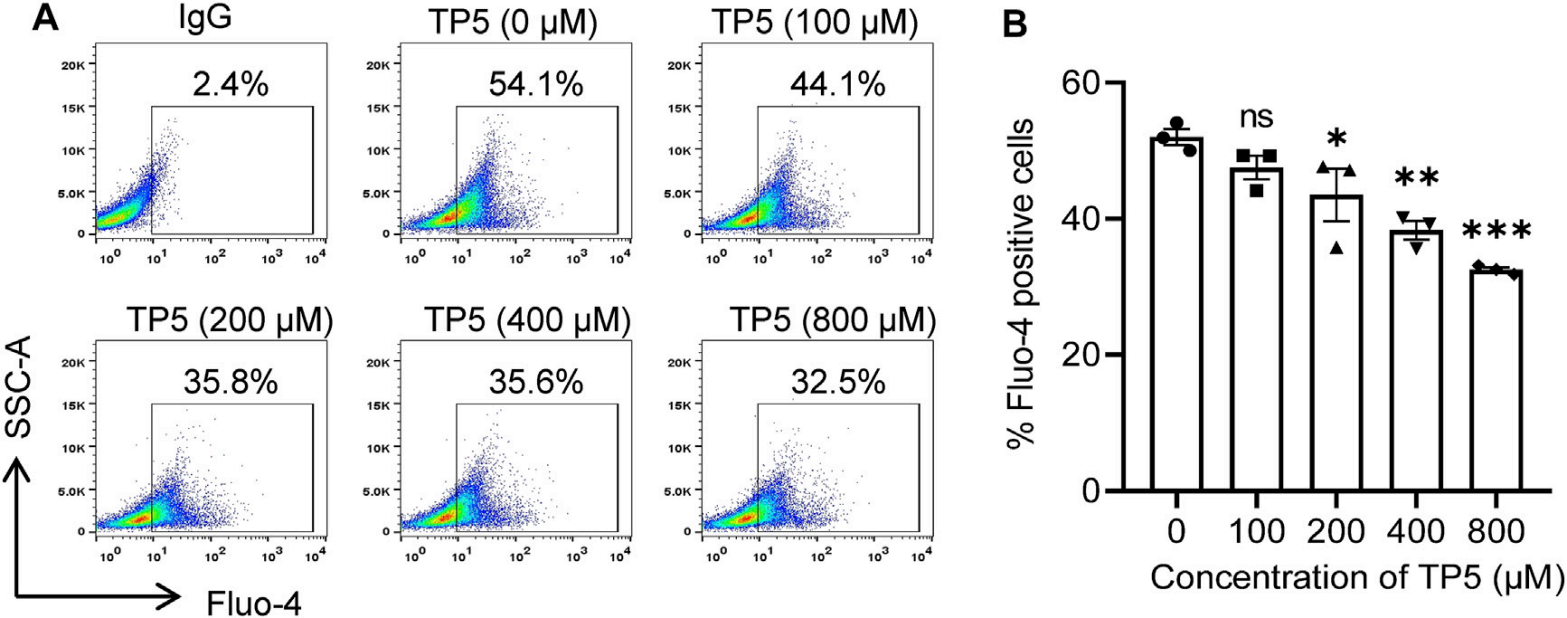
TP5 reduces intracellular calcium concentration in HCT116 cells. **(A, B)** Representative cellular calcium decrease **(A)** and the summary of three independent experiments **(B)**. Altered intracellular calcium concentration of HCT116 cells after various concentrations of TP5 treatment. All data are expressed as mean ± SD; ^*^ *p* < 0.05, ^**^ *p* < 0.01 and ^***^ *p* < 0.001, one-way ANOVA with Dunnett’s post hoc test [F (4, 10) = 13.81, *p* < 0.001]; ns, not significant.

As a universal second messenger, calcium ions (Ca^2+^) promote the growth and metastasis of certain malignant tumor cells (Roderick and Cook, 2008; Yang et al., 2021). The self-renewal, drug resistance, and tumorigenic properties of colon cancer stem cells may function by regulating the inward flow of Ca^2+^ (Terrie et al., 2019); Ca^2+^ can promote the growth and spread of cancer stem cells. The percentage of Fluo-4 positive cells decreased from 52.0 ± 2.1% to 47.5 ± 3.0%, 43.5 ± 6.7%, 38.3 ± 2.4% and 32.5 ± 0.6% after 24 h of action of TP5 on HCT116 cells with 100, 200, 400 and 800 μM, respectively (Figures 5A,B), indicating that TP5 can concentration dependently reduce the intracellular Ca^2+^ concentration of HCT116 cells and thus inhibit the properties of cancer stem cells.

### TP5 Promotes the Anti-proliferative Effect of OXA on Colon Cancer Cells

**FIGURE 6:**
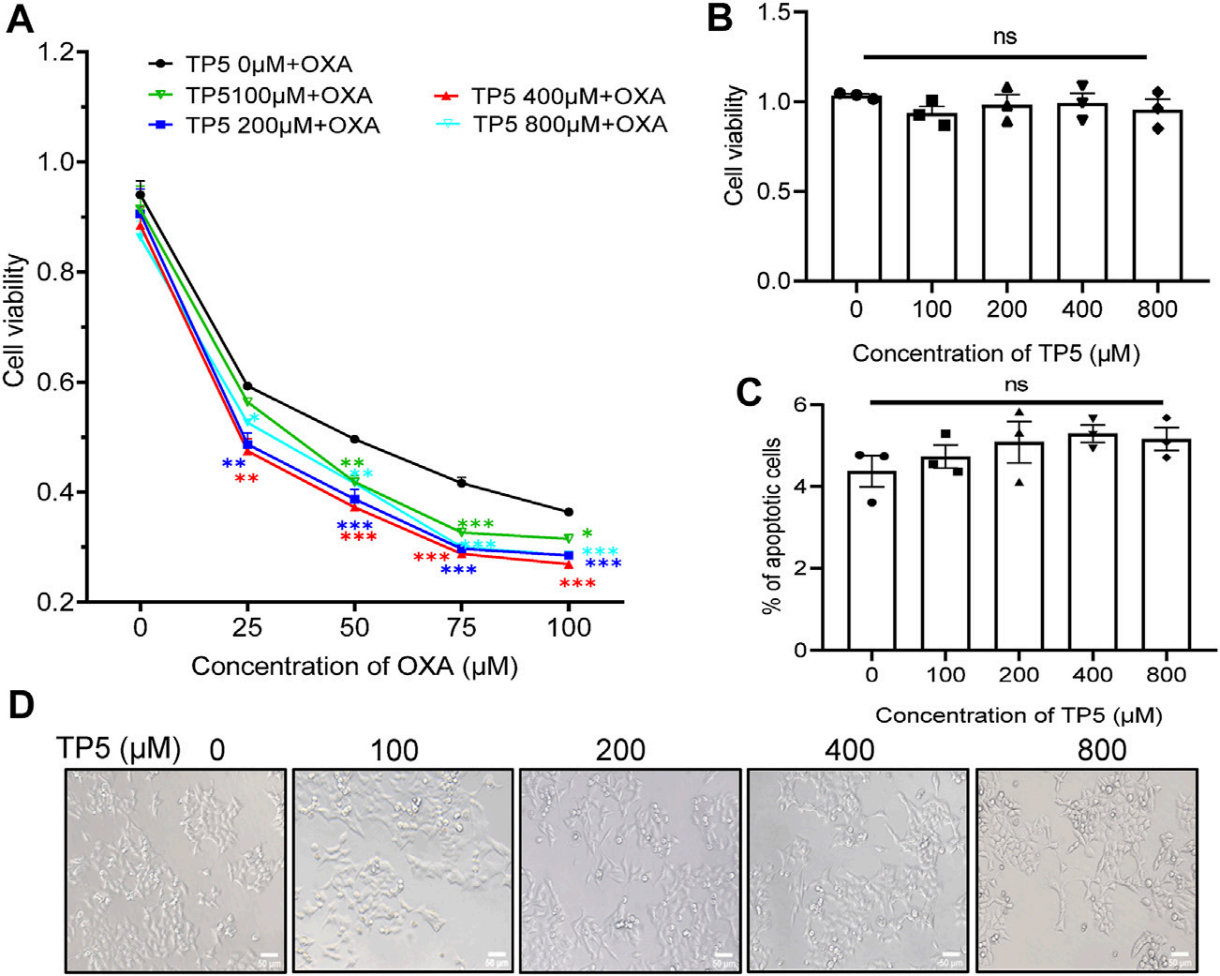
TP5 promotes the anti-proliferative effect of OXA on colon cancer cells. **(A)** The HCT116 cell viability measured by MTT assay at different concentrations of TP5 and OXA. HCT116 cells were exposed to different concentrations of TP5 (0, 100, 200, 400, and 800 μM) and OXA (0, 25, 50, 75, and 100 μM) for 48 h. **(B)** The HCT116 cell viability measured by MTT assay after treatment with TP5 for 48 h only. **(C)** Apoptosis of HCT116 cells analyzed by Annexin V/PI staining by flow cytometry after treatment with different concentrations of TP5. **(D)** Morphological changes observed in TP5-treated HCT116 cells and untreated cells. Scale bar = 50 μm. All data are expressed as mean ± SD; ^*^ *p* < 0.05, ^**^ *p* < 0.01 and ^***^ *p* < 0.001, two-way ANOVA with Dunnett’s post hoc test **(A)**, F (4, 50) = 25.15, *p* < 0.0001, and one-way ANOVA with Dunnett’s post hoc test **(B)** and **(C)**, F (4, 10) = 0.6217, *p* = 0.6575, **(C)**, F (4, 10) = 1.132, *p* = 0.3952; ns, not significant.

Given the possible resistance of cancer stem cells to chemotherapeutic agents (Prieto-Vila et al., 2017) and the diminished proliferation inhibition of tumor cells by chemotherapeutic agents, the direct inhibitory effect of TP5 on colon cancer stem cells suggests that the combination of TP5 with chemotherapeutic agents may enhance the therapeutic effect of these chemotherapeutic agents. Consistent with this speculation, we show that 25–100 μM of chemotherapeutic drug OXA produced inhibition on the proliferation of HCT116 in a concentration-dependent manner, while 100–800 μM TP5 significantly enhanced the anti-proliferative effect of OXA (Figure 6A).

To rule out the possibility that TP5 significantly enhanced the anti-proliferative effect of OXA on HCT116 because TP5 directly inhibited the proliferation of HCT116 cells, we treated HCT116 cells with TP5 for 48 h and observed its inhibitory effect on the proliferation of HCT116.100–800 μM TP5 did not cause significant visible changes in the cell viability and morphology of HCT116 cells compared with the control (Figures 6B,D); also, 100–800 μM TP5 did not induce apoptosis in HCT116 cells (Figure 6C). These results suggested that TP5 did not have a direct effect on the proliferation, morphology, and apoptosis of HCT116 cells.

**FIGURE 7:**
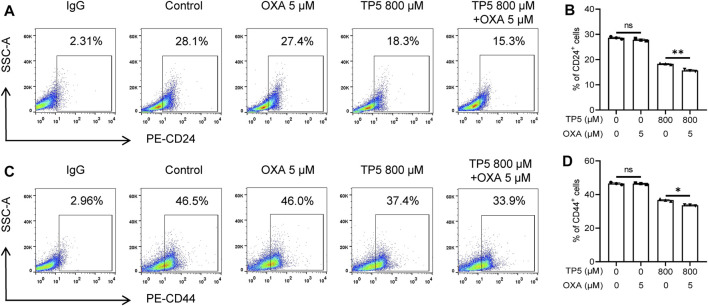
TP5 promotes the inhibitory effect of OXA on colon cancer stem cells. **(A–D)** Flow cytometer analysis of the expression of CD24 **(A)** and CD44 **(C)** in HCT116 cells after OXA and TP5 treatments, and pooled data of cell surface markers CD24 **(B)**, CD44 **(D)** (*n* = 3 independent experiments). All data are represented as mean ± SD; ^*^ *p* < 0.05 and ^**^ *p* < 0.01, unpaired two-tailed Student’s *t*-test. ns, not significant.

To further verify whether TP5 promotes the antitumor effect of OXA by inhibiting colon cancer stem cells, we measured the expression of cancer stem cell markers CD24 and CD44 by flow cytometry after TP5 and OXA were co-administered to colon cancer HCT116 cells for 24 h. Compared with the control group, 5 μM OXA could not change the proportion of CD24-positive cells (28.6 ± 0.5% vs 27.8 ± 0.5%, *p* > 0.05; Figures 7A,B), while the proportion of CD24-positive cells decreased from 28.6 ± 0.5% to 18.3 ± 0.3% after 800 μM TP5 was administered to HCT116 cells for 24 h (*p* < 0.01, Figures 7A,B). Co-administration of TP5 (800 μM) with OXA (5 μM) further reduced the proportion of CD24-positive cells to 15.7 ± 0.4% (*p* < 0.01, Figures 7A,B). Similarly, 5 μM OXA could not change the proportion of CD44-positive cells, while the proportion of CD44-positive cells decreased from 46.6 ± 0.7% to 36.7 ± 0.7% after 24 h of 800 μM TP5 action on HCT116 cells, and the combined administration of TP5 (800 μM) with OXA (75 μM) further reduced the proportion of CD44-positive cells proportion to 33.6 ± 0.4% (*p* < 0.01, Figures 7C,D). It suggests that TP5 may inhibit colon cancer stem cells and therefore possibly sensitize the antitumor effect of OXA.

### TP5 Inhibits HCT116 Cancer Stem Cells *via* Acetylcholine Receptors (nAchRs)

**FIGURE 8:**
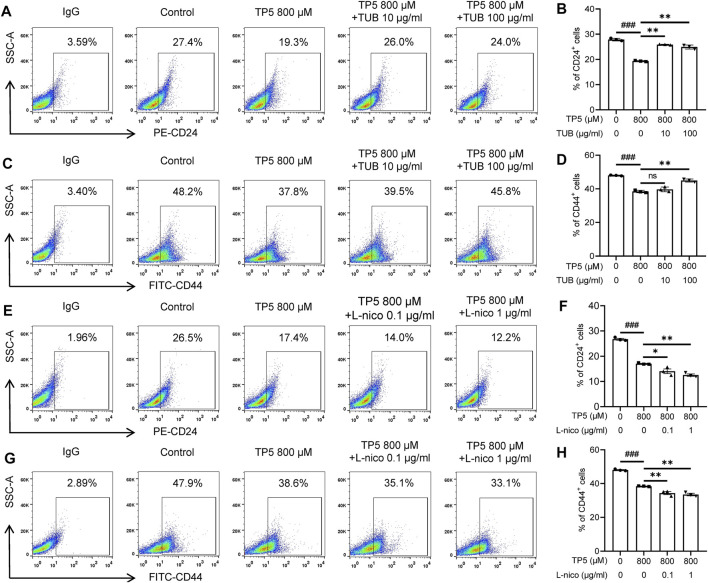
TP5 inhibits HCT116 cancer stem cells via acetylcholine receptors (AchRs). **(A–D)** Flow cytometric analysis of CSC surface markers CD24 **(A, B)** and CD44 **(C, D)** in HCT116 cells treated with TP5-conjugated AchR inhibitor D-Tubocurarine (TUB). **(E–H)** Flow cytometric analysis of CSC surface markers CD24 **(E, F)** and CD44 **(G, H)** in HCT116 cells treated with TP5-conjugated AchR agonist L-nicotine (L-Nico). Data are represented as mean ± SD of three independent experiments; ^*^ *p* < 0.05, ^**^ *p* < 0.01 and ^***^ *p* < 0.001, one-way ANOVA with Tukey’s post hoc test, **(A)**, F (3, 8) = 158.6, *p* < 0.0001, **(B)**, F (3, 8) = 71.08, *p* < 0.0001, **(C)**, F (3, 8) = 123.9, *p* < 0.0001, **(D)**, F (3, 8) = 120.8, *p* < 0.0001; ns, not significant.

Our previous studies suggested that D-tubocurarine (TUB), an antagonist of nAchRs (Fan et al., 2006b), significantly antagonized the inhibitory effect of TP5 on the proliferation of leukemic HL60 cells, whereas the inhibitor of muscarinic acetylcholine receptors (mAchRs), atropine (Atr), did not have this antagonistic effect, suggesting that the inhibitory proliferative effect of TP5 on leukemic cells may be achieved through nAchRs (Fan et al., 2006a). We further investigated whether TP5 inhibited nAchRs via acetylcholine receptors in HCT116 colon cancer stem cells by using agonists and antagonists of nAchRs. TUB (10–100 μg/ml) significantly antagonized this effect of TP5 (Figures 8A–D). After 24 h of TP5 action on HCT116 cells with 800 μM, the percentage of CD24-positive cells decreased from 27.9 ± 0.5% to 19.3 ± 0.3% (*p* < 0.01) and the percentage of CD44-positive cells decreased from 48.0 ± 0.3% to 38.4 ± 0.7% (*p* < 0.001); whereas the early addition of 10 μg/ml or 100 μg/ml TUB, the proportion of CD24-positive cells reversed to 25.9 ± 0.2% (*p* < 0.01, vs. 19.3 ± 0.3%) and 24.9 ± 0.8% (*p* < 0.01, vs. 19.3 ± 0.3%); the proportion of CD44-positive cells reversed to 39.7 ± 1.4% (*p* > 0.05 vs. 38.4 ± 0.7%) and 45.0 ± 0.9% (*p* < 0.01 vs. 38.4 ± 0.7%).

In contrast, the cholinergic agonist the nicotine L-Nicotine (L-Nico) further promoted the inhibitory effect of TP5 on cancer stem cells in colon cancer cells (Figures 8E–H). Pretreatments of L-Nico (0.1 and 1 μg/ml) further reduced the proportion of CD24-positive cells to 14.0 ± 1.7%, 12.5 ± 0.9% (*p* < 0.01, vs. 19.3 ± 0.3%), and the proportion of CD44-positive cells to 34.3 ± 1.6%, and 33.5 ± 1.2% (*p* < 0.05 vs. 38.4 ± 0.7%).

**FIGURE 9:**
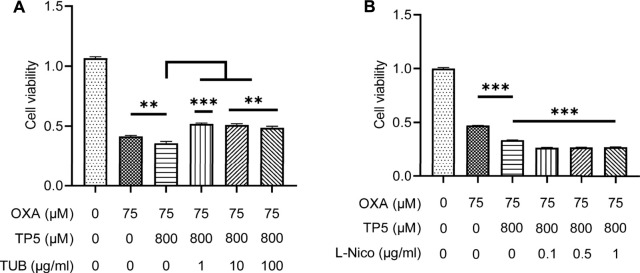
TP5 promotes the inhibitory effect of OXA on colon cancer cell proliferation *via* AchRs. **(A, B)** HCT116 cells were tested for cell viability after treatment with TP5 and OXA, or in combination with AchR inhibitors TUB (1, 10 and 100 μg/ml) **(A)**, or with AchR agonists L-Nico (0.1, 0.5 and 1 μg/ml) **(B)** for 48 h. All data are represented as mean ± SD. ^**^ *p* < 0.01, ^***^ *p* < 0.001, unpaired two-tailed Student’s *t*-test; ns, not significant.

We further found that the inhibitor TUB (1–100 μg/ml) significantly blocked the TP5-promoted proliferation inhibition of HCT116 by OXA, whereas the agonist L-Nico promoted this effect of TP5 (Figure 9A, B), implying that nAchRs might be involved in TP5-mediated inhibition of HCT116 colon cancer stem cells inhibition, thereby interrupting the TP5-promoted proliferation inhibition of HCT116 by oxaliplatin. Certainly, since restoration of cell viability in the presence of TP5 is only partial, there may be other mechanisms for TP5 to act on HCT116 cells in addition to nAchRs.

## Discussion

According to a large number of clinical studies (Costantini et al., 2019), TP5 is indicated for immune impairment due to malignant tumors and therefore can be used frequently in tumor patients and is very safe, with very few toxic side effects, both subcutaneously and intravenously (Xiaojing et al., 2017). In the present study, we found that the immunomodulatory peptide TP5 can not only be used as a good immune adjuvant in the treatment of tumors, but also can act directly on colon cancer cells. TP5 could not inhibit the proliferation of colon cancer cells HCT116, but it could enhance the anti-proliferative effect of chemotherapeutic drug oxaliplatin on colon cancer cells HCT116.

Evidence from the following aspects suggests that TP5 can effectively influence the stemness of HCT116 cancer stem cells. *First*, the ability of sphere formation is an important method to identify cancer stem cells *in vitro* (Cao et al., 2011). After TP5 action, the number of cancer stem cell spheroids formed by HCT116 cells was significantly reduced, and the spheroid formation rate was also significantly decreased, indicating that TP5 inhibited the self-renewal and proliferation ability of cancer stem cells. *Secondly*, CD24, CD44 and CD133 are specific surface markers that can be expressed by colon cancer stem cells (Choi et al., 2009); ALDH1, OCT4, SOX2 and Nanog are all markers of stemness status of cancer stem cells, which are important for maintaining the nature and drug resistance of cancer stem cells (Liu et al., 2013). TP5 significantly decreased the expression of these important stemness genes, thus inhibiting stemness of colon cancer stem cells. *Third*, the self-renewal, drug resistance and tumorigenicity of colon cancer stem cells may function by regulating the inward flow of Ca^2+^ (Yang et al., 2020). Ca^2+^ promote the growth and proliferation of cancer stem cells. TP5 can concentration-dependently decrease the intracellular calcium ion concentration in HCT116 cells, thus inhibiting the properties of cancer stem cells. *Fourth*, the wnt/β-catenin signaling pathway is a key pathway for cancer stem cell development (Valkenburg et al., 2011). Sustained activation of the wnt signaling pathway leads to the generation of cancer stem cells, thus blocking the wnt/β-catenin signaling pathway may be the key to removing cancer stem cells for the treatment of colon cancer (Krishnamurthy and Kurzrock, 2018). TP5 can inhibit cancer stem cells by inhibiting the PI3K/Akt/wnt/β-catenin signaling pathway to inhibit cancer stem cells, thereby suppressing colon cancer stem cells.

The mechanism by which TP5 enhances the antitumor effect of the chemotherapeutic drug OXA by affecting the stemness of HCT116 cancer stem cells may be as follows. 1. Cancer stem cells have an enhanced ability to repair DNA damage, which induces resistance to chemotherapy, including resistance to DNA-damaging drugs (Yu et al., 2017). TP5 inhibits colon cancer stem cells and reduces the ability of cancer stem cells to repair DNA damage, thereby enhancing stem cell sensitivity to the DNA-damaging drug OXA. 2. Cancer stem cells, like stem cells, can be more viable through multiple mechanisms that can avoid cell death due to apoptosis and senescence (Sabisz and Skladanowski, 2009; Triana-Martinez et al., 2020). TP5 can inhibit colon cancer stem cells, thus reducing the viability of stem cells and thus enhancing the cytotoxicity of chemotherapeutic drugs on cancer stem cells. 3. TP5 and OXA target both HCT116 tumor cells and stem cells, producing a synergistic effect. TP5 acts mainly on cancer stem cells and OXA acts mainly on tumor cells. However, OXA also reduced the expression of CD24 and CD44, which are specific surface markers for colon cancer stem cells, while TP5 mildly enabled OXA to exert an inhibitory effect on stem cell stemness. Thus, the synergistic effect between the two ultimately enhanced the antitumor effect of OXA.

Colorectal cancer is the third most common cancer and the fourth most common cause of death in the world, with approximately 700,000 deaths worldwide each year (Rawla et al., 2019). The development and metastasis of cancer is thought to be caused by cancer stem cells. Cancer stem cells are latent in the body, ready to metastasize and cause tumor recurrence in a more aggressive and drug-resistant form (Chang, 2016). Colon cancer stem cells are highly resistant to radiotherapy and chemotherapy, and most drugs can act on tumor cells but are ineffective against cancer stem cells. Therefore, inhibition of cancer stem cells is a critical and difficult point in tumor treatment (Ordonez-Moran et al., 2015; Hirata et al., 2019). TP5 can promote the antitumor effect of oxaliplatin by inhibiting colon cancer stem cells, suggesting that TP5 may become a novel drug to target cancer stem cells, thus reducing cancer recurrence rate and improving tumor treatment efficacy, and may provide a new treatment option for patients with drug-refractory colorectal cancer. Nevertheless, many questions are still unclear: here, can TP5 inhibit colon cancer stem cells, however, does TP5 have any effect on other types of cancer stem cells, and does TP5 act directly on acetylcholine receptors.

Finally, for the adjuvant treatment of TP5, the dose is mainly intramuscular or subcutaneous and may be 50 mg per day (Conant et al., 1992; Fabrizi et al., 2006; Wolf et al., 2011). One study evaluated the clinical efficacy and tolerability of high-dose intravenous TP5 in 16 patients with melanoma (Cascinelli et al., 1998). Patients received 1 g of intravenous TP5 every 2 days for 7 weeks and were then evaluated; responders received a follow-up course of 2 g of intravenous TP5 every 2 days for 5 weeks. In this study, high-dose intravenous TP5 administered three times weekly enhances immune function in patients with cutaneous and subcutaneous metastases from melanoma without associated side effects (Cascinelli N et al., 1998). Therefore, TP5 may reach relatively high concentrations at the local administration site, but the concentrations in the whole blood of patients may be lower than in our experiments. This study might not directly guide the clinical application of TP5 at this time, but offers the possibility of continuing to modify the TP5 sequence to increase its potency to act on cancer stem cells and protect against immune repair, such as through some unnatural amino acid substitutions, cyclization modifications, etc. Therefore, further research on these scientific questions may lay a solid theoretical foundation for the realization of immunomodulatory peptide TP5/TP5 analogs alone or in combination with chemotherapeutic drugs for the clinical treatment of colon cancer.

## Materials and Methods

### Chemicals and Cell Culture

TP5 was purchased from ChemBest Research Laboratories Ltd. (China). Oxaliplatin (OXA), D-tufosine pentahydrate (TUB) and L-nicotine (L-Nico) were purchased from MedChemExpress (USA). HCT116 cells were cultured in RPMI-1640 medium (C11875500BT, Gibco) supplemented with 10% fetal bovine serum (10099–141, Gibco), penicillin (100 IU/ml) and streptomycin (100 μg/ml, C0222, Beyotime). LoVo cells were cultured in F-12K Nutrient Mixture (21127–022, Gibco) supplemented with 10% fetal bovine serum (10099–141, Gibco), penicillin (100 IU/ml) and streptomycin (100 μg/ml, C0222, Beyotime). Cells were incubated in a humidified incubator containing 5% CO_2_ at 37°C.

### Sphere Formation Test

The serum-free medium for spheroid culture consisted of DMEM/F12 (SH30023.01, HyClone) medium supplemented with 0.4% BSA (B2064-100G, Sigma), 20 ng/ml human recombinant epidermal growth factor (AF-100-15-100UG, PeproTech), 20 ng/ml human recombinant basic fibroblast growth factor (100–18 B-10UG, PeproTech), 25 μg/ml human insulin (HY-P0035, MCE), 2% B27 supplement (50×) (17504–044, Gibco), and 0.1% 2-mercaptoethanol (21985023, Gibco). Cells were plated in Corning Ultra-Low Attachment 24-well plates (3473, Costar) at a density of 5,000 viable cells/well and grown in serum-free medium. Cells were grown for 6 days and the number of spheroids was counted under the microscope (DMI3000 B, Leica) as previously described (Tominaga et al., 2019).

### Flow Cytometry

HCT116 cells or LoVo cells (1.5 × 10^5^ cells/well) were seeded in 12-well plates and exposed to different concentrations of TP5 for 24 h. The supernatant was then removed and cells were digested with 0.25% trypsin (25200-072, Gibco). After washing, cells were resuspended in 100 ul of PBS (ST476, Beyotime). For surface staining, cells were labeled with PE-CD24 (555428, BD Pharmingen), FITC-CD44 (555478, BD Pharmingen), and PE-CD133 (12-1338-41, eBiosciences) (He et al., 2021). Cell suspensions were incubated with the appropriate antibodies for 30 min at room temperature in the dark, followed by a washing step to remove unlabeled antibodies. Flow cytometry analysis was performed using BD FACS Jazz™ (BD Biosciences) and analyzed by FlowJo software.

### RT—qPCR Assay

Total RNA was isolated and reverse transcribed using EZ-press RNA purification kit (B0004-plus, EZBioscience) and PrimeScript RT kit (RR047A, TaKaRa), respectively, according to the manufacturer’s instructions (Wang et al., 2013), and qPCR was performed using TB Green Premix Ex Taq (RR420A. TaKaRa) for qPCR. relative changes in gene expression were determined using the 2-ΔΔCt method, and relative mRNA expression was normalized to GAPDH. Following primer sets were used for analyzing the expression of specific genes including ALDH1, forward: 5′-ACG​CCA​GAC​TTA​CCT​GTC​CTA​CTC-3′ and reverse: 5′- TCT​TGC​CAC​TCA​CTG​AAT​CAT​GCC-3′; SOX-2, forward: 5′- GCT​CGC​AGA​CCT​ACA​TGA​ACG​G-3′, and reverse: 5′-AGC​TGG​CCT​CGG​ACT​TGA​CC -3′; OCT-4, forward: 5′- GATGTGGTCCGAGTGTGGTTCTG-3′and reverse: 5′-CGA​GGA​GTA​CAG​TGC​AGT​GAA​GTG -3′; Nanog, forward: 5′- AGC​AAT​GGT​GTG​ACG​CAG​AAG​G-3′, and reverse: 5′-ACC​AGG​TCT​GAG​TGT​TCC​AGG​AG -3′; CD24, forward: 5′- CTC​CTA​CCC​ACG​CAG​ATT​TAT​TC-3′, and reverse: 5′-AGA​GTG​AGA​CCA​CGA​AGA​GAC-3′; CD44, forward: 5′- CTG​CCG​CTT​TGC​AGG​TGT​A-3′, and reverse: 5′-CAT​TGT​GGG​CAA​GGT​GCT​ATT-3′; CD133, forward: 5′- AGT​CGG​AAA​CTG​GCA​GAT​AGC-3′, and reverse: 5′-GGT​AGT​GTT​GTA​CTG​GGC​CAA​T-3′; GAPDH, forward: 5′-TTG​GTA​TCG​TGG​AAG​GAC​T-3′, and reverse: 5′-GGA​TGA​TGT​TCT​GGA​GAG​C-3′.

### Aldehyde Dehydrogenase Activity Assay

ALDH activity was assessed using an ALDH activity detection kit (BC0755, Solarbio) according to the manufacturer’s instructions (Jiang et al., 2019). HCT116 cells (3.5 × 10^5^ cells/well) were seeded in 6-well plates with different treatments of TP5 (0, 100, 200, 400, and 800 μM) for 48 h at 37°C. Cells were lysed with ultrasound, centrifuged and the supernatant (100 μL/well) was transferred to UV-Star 96-well plates (655801, Greiner Bio-one) and incubated at 37°C for 10 min. ALDH activity was measured at 340 nm using a microplate reader (VarioskanTM LUX, Thermo).

### Western Blot Analysis

After treatment with TP5 (100, 200, 400 and 800 µM) for 48 h, total proteins from HCT116 cells were lysed in lysis buffer (P0013, Beyotime) containing 1% cocktail (B14001, Bimake) (Li et al., 2012; Wang et al., 2017). Cell lysates were cleared by centrifugation at 12,000 rpm for 15 min at 4°C. The concentration of total protein was determined using the Enhanced BCA Protein Assay Kit (P0010, Beyotime). Protein sample buffer was added and samples were boiled at 100°C for 10 min. Briefly, 20 µg of protein was loaded into each lane and transferred to a polyvinylidene difluoride (PVDF) membrane. The membranes were incubated overnight at 4°C with primary antibody, and secondary antibodies anti-rabbit IgG HRP-conjugated (1: 3,000, 7074S, Cell Signaling Technology) and anti-mouse IgG HRP-conjugated (1: 3,000, 7076S, Cell Signaling Technology) were incubated at 25°C for incubate for 2 h. The primary antibodies used are Beta Catenin Rabbit Polyclonal Antibody (1: 1,000, 51067-2-AP, Proteintech), WNT1 Rabbit Polyclonal Antibody (1: 1,000, 27935-1-AP, Proteintech), Phospho-beta Catenin (Ser33/37/Thr41) antibody (1: 1,000, 9561, Cell Signaling Technology), AKT1 (Phospho-Ser473 + Tyr474) antibody (1: 1,000, 12669, Abcam), PI3K p110 (beta) polyclonal antibody (1: 1,000, 20584-1-AP, Proteintech), AKT1 polyclonal antibody (1: 1,000, 10176-2-AP, Proteintech), and GAPDH (1: 10,000, 60004-I-Ig, Proteintech). Phosphorylated protein bands were categorized as their total protein level, and other bands were categorized as GAPDH.

### Molecular Probes Fluo-4 AM Calcium Assay

HCT116 cells were incubated in 12-well plates with different treatments of TP5 (0, 100, 200, 400, 800 μM) for 24 h at 37°C. To determine the cellular calcium concentration (He et al., 2021), the cell membrane Ca^2+^-free concentration was assessed using the fluorescent Ca^2+^-probe Fluo-4 AM (S1060, Beyotime). Briefly, cells were incubated in PBS at 37°C with 2 μM Fluo-4 AM for 30 min in the dark, followed by three washes with PBS. Binding buffer (500 μL) containing 1% FBS (Gibco, Australia) was added and incubated for 30 min in the dark at room temperature. The results were then evaluated by flow cytometry. The excitation wavelength was 488 nm and the emission wavelength was 520 nm. The experiment was repeated at least three times.

### Cell Viability Assay

Cell viability was assessed using the MTT assay kit (C0009, Beyotime) according to the manufacturer’s protocol (Foldbjerg et al., 2011). HCT116 cells were seeded in 96-well plates at a density of 15 × 103 cells/well and incubated overnight at 37°C. Next, cells were treated with TP5 and OXA and incubated for 48 h 10 μl MTT was added to each well and incubation was continued for 4 h. The absorbance was measured at 570 nm using a microplate reader (VarioskanTM LUX, Thermo). Cell viability was estimated by comparing the relative absorbance values with those of the untreated samples.

### Annexin V/Propidium Iodide Staining Assay

The extent of apoptosis in treated HCT116 cells was assessed using the Annexin V-FITC/PI Apoptosis Assay Kit (556547, BD Pharmingen) according to the manufacturer’s instructions (Park et al., 2017). HCT116 cells (1.5 × 10^5^ cells/well) were seeded in 12-well plates, exposed to different concentrations of TP5 for 48 h, and stained with 5 μl of FITC-labeled Annexin V and 5 μl of PI simultaneously at room temperature, protected from light. Stained cells were analyzed using a fluorescence-activated cell sorter flow cytometer (BD FACS Jazz™, BD Biosciences). A minimum of 10,000 cells were used for each analysis, and experiments were performed in triplicate.

### Statistical Analysis

Data are expressed as mean ± SD. All experiments were performed independently, at least 3 times. Statistical analyses were performed as described in each corresponding legend. Differences between two groups were assessed by unpaired two-sided Student’s t-test, and differences between multiple groups were assessed by one- or two-way ANOVA and Dunnett’s post hoc test. *p* less than 0.05 was considered statistically significant.

## Data Availability

The original contributions presented in the study are included in the article/Supplementary Material, further inquiries can be directed to the corresponding authors.

## References

[B1] Anorma C. Hedhli J. Bearrood T. E. Pino N. W. Gardner S. H. Inaba H. (2018). Surveillance of Cancer Stem Cell Plasticity Using an Isoform-Selective Fluorescent Probe for Aldehyde Dehydrogenase 1A1. ACS Cent. Sci. 4 (8), 1045–1055. 10.1021/acscentsci.8b00313 30159402 PMC6107868

[B2] Ayob A. Z. Ramasamy T. S. (2018). Cancer Stem Cells as Key Drivers of Tumour Progression. J. Biomed. Sci. 25 (1), 20–18. 10.1186/s12929-018-0426-4 29506506 PMC5838954

[B3] Bocci F. Gearhart-Serna L. Boareto M. Ribeiro M. Ben-Jacob E. Devi G. R. (2019). Toward Understanding Cancer Stem Cell Heterogeneity in the Tumor Microenvironment. Proc. Natl. Acad. Sci. U S A. 116 (1), 148–157. 10.1073/pnas.1815345116 30587589 PMC6320545

[B4] Bodeya B. Bodeya B. jr Siegel S. E. Kaiser H. E. (2000). Review of Thymic Hormones in Cancer Diagnosis and Treatment. Int. J. Immunopharmacol. 22, 261–273. 10.1016/s0192-0561(99)00084-3 10689100

[B5] Cao L. Zhou Y. Zhai B. Liao J. Xu W. Zhang R. (2011). Sphere-forming Cell Subpopulations with Cancer Stem Cell Properties in Human Hepatoma Cell Lines. BMC Gastroenterol. 11, 71. 10.1186/1471-230X-11-71 21669008 PMC3136412

[B6] Cascinelli N. Belli F. Mascheroni L. Lenisa L. Clemente C. (1998). Evaluation of Clinical Efficacy and Tolerability of Intravenous High Dose Thymopentin in Advanced Melanoma Patients. Melanoma Res. 8 (1), 83–89. 10.1097/00008390-199802000-00014 9508382

[B7] Chambers I. Colby D. Robertson M. Nichols J. Lee S. Tweedie S. (2003). Functional Expression Cloning of Nanog, a Pluripotency Sustaining Factor in Embryonic Stem Cells. Cell 113 (5), 643–655. 10.1016/s0092-8674(03)00392-1 12787505

[B8] Chang J. C. (2016). Cancer Stem Cells: Role in Tumor Growth, Recurrence, Metastasis, and Treatment Resistance. Medicine (Baltimore) 95 (1 Suppl. 1), S20–S25. 10.1097/MD.0000000000004766 27611935 PMC5599212

[B9] Choi D. Lee H. W. Hur K. Y. Kim J. J. Park G. S. Jang S. H. (2009). Cancer Stem Cell Markers CD133 and CD24 Correlate with Invasiveness and Differentiation in Colorectal Adenocarcinoma. World J. Gastroenterol. 15 (18), 2258–2264. 10.3748/wjg.15.2258 19437567 PMC2682242

[B10] Ciombor K. K. Wu C. Goldberg R. M. (2015). Recent Therapeutic Advances in the Treatment of Colorectal Cancer. Annu. Rev. Med. 66, 83–95. 10.1146/annurev-med-051513-102539 25341011

[B11] Clara J. A. Monge C. Yang Y. Takebe N. (2020). Targeting Signalling Pathways and the Immune Microenvironment of Cancer Stem Cells - a Clinical Update. Nat. Rev. Clin. Oncol. 17 (4), 204–232. 10.1038/s41571-019-0293-2 31792354

[B12] Conant M. A. Calabrese L. H. Thompson S. E. Poiesz B. J. Rasheed S. Hirsch R. L. (1992). Maintenance of CD4+ Cells by Thymopentin in Asymptomatic HIV-Infected Subjects: Results of a Double-Blind, Placebo-Controlled Study. AIDS 6 (11), 1335–1339. 10.1097/00002030-199211000-00016 1361746

[B13] Costantini C. Bellet M. M. Pariano M. Renga G. Stincardini C. Goldstein A. L. (2019). A Reappraisal of Thymosin Alpha1 in Cancer Therapy. Front. Oncol. 9, 873. 10.3389/fonc.2019.00873 31555601 PMC6742685

[B14] Csaba G. (2016). The Immunoendocrine Thymus as a Pacemaker of Lifespan. Acta Microbiol. Immunol. Hung 63 (2), 139–158. 10.1556/030.63.2016.2.1 27352969

[B15] Du L. Wang H. He L. Zhang J. Ni B. Wang X. (2008). CD44 Is of Functional Importance for Colorectal Cancer Stem Cells. Clin. Cancer Res. 14 (21), 6751–6760. 10.1158/1078-0432.CCR-08-1034 18980968

[B16] Fabrizi F. Dixit V. Martin P. (2006). Meta-analysis: the Adjuvant Role of Thymopentin on Immunological Response to Hepatitis B Virus Vaccine in End-Stage Renal Disease. Aliment. Pharmacol. Ther. 23 (11), 1559–1566. 10.1111/j.1365-2036.2006.02923.x 16696803

[B17] Fan Y. Z. Chang H. Yu Y. Liu J. Wang R. (2006a). Thymosin Alpha1 Suppresses Proliferation and Induces Apoptosis in Human Leukemia Cell Lines. Peptides 27 (9), 2165–2173. 10.1016/j.peptides.2006.03.012 16644063

[B18] Fan Y. Z. Chang H. Yu Y. Liu J. Zhao L. Yang D. J. (2006b). Thymopentin (TP5), an Immunomodulatory Peptide, Suppresses Proliferation and Induces Differentiation in HL-60 Cells. Biochim. Biophys. Acta 1763 (10), 1059–1066. 10.1016/j.bbamcr.2006.07.004 16952408

[B19] Fearon E. R. Carethers J. M. (2015). Molecular Subtyping of Colorectal Cancer: Time to Explore Both Intertumoral and Intratumoral Heterogeneity to Evaluate Patient Outcome. Gastroenterology 148 (1), 10–13. 10.1053/j.gastro.2014.11.024 25451650 PMC4471844

[B20] Foldbjerg R. Dang D. A. Autrup H. (2011). Cytotoxicity and Genotoxicity of Silver Nanoparticles in the Human Lung Cancer Cell Line, A549. Arch. Toxicol. 85 (7), 743–750. 10.1007/s00204-010-0545-5 20428844

[B21] Hammond W. A. Swaika A. Mody K. (2016). Pharmacologic Resistance in Colorectal Cancer: a Review. Ther. Adv. Med. Oncol. 8 (1), 57–84. 10.1177/1758834015614530 26753006 PMC4699262

[B22] He X. Wan J. Yang X. Zhang X. Huang D. Li X. (2021). Bone Marrow Niche ATP Levels Determine Leukemia-Initiating Cell Activity via P2X7 in Leukemic Models. J. Clin. Invest. 131 (4), e140242. 10.1172/JCI140242 PMC7880412 33301426

[B23] Hervieu C. Christou N. Battu S. Mathonnet M. (2021). The Role of Cancer Stem Cells in Colorectal Cancer: From the Basics to Novel Clinical Trials. Cancers (Basel) 13 (5), 1092. 10.3390/cancers13051092 33806312 PMC7961892

[B24] Hirata A. Hatano Y. Niwa M. Hara A. Tomita H. (2019). Heterogeneity of Colon Cancer Stem Cells. Adv. Exp. Med. Biol. 1139, 115–126. 10.1007/978-3-030-14366-4_7 31134498

[B25] Jaggupilli A. Elkord E. (2012). Significance of CD44 and CD24 as Cancer Stem Cell Markers: an Enduring Ambiguity. Clin. Dev. Immunol. 2012, 708036. 10.1155/2012/708036 22693526 PMC3369436

[B26] Jiang T. Zhao J. Yu S. Mao Z. Gao C. Zhu Y. (2019). Untangling the Response of Bone Tumor Cells and Bone Forming Cells to Matrix Stiffness and Adhesion Ligand Density by Means of Hydrogels. Biomaterials 188, 130–143. 10.1016/j.biomaterials.2018.10.015 30343256 PMC6279509

[B27] Kreso A. Dick J. E. (2014). Evolution of the Cancer Stem Cell Model. Cell Stem Cell 14 (3), 275–291. 10.1016/j.stem.2014.02.006 24607403

[B28] Krishnamurthy N. Kurzrock R. (2018). Targeting the Wnt/Beta-Catenin Pathway in Cancer: Update on Effectors and Inhibitors. Cancer Treat. Rev. 62, 50–60. 10.1016/j.ctrv.2017.11.002 29169144 PMC5745276

[B29] Li J. Liu C. H. Wang F. S. (2010). Thymosin Alpha 1: Biological Activities, Applications and Genetic Engineering Production. Peptides 31 (11), 2151–2158. 10.1016/j.peptides.2010.07.026 20699109 PMC7115394

[B30] Li V. S. Ng S. S. Boersema P. J. Low T. Y. Karthaus W. R. Gerlach J. P. (2012). Wnt Signaling through Inhibition of β-catenin Degradation in an Intact Axin1 Complex. Cell 149 (6), 1245–1256. 10.1016/j.cell.2012.05.002 22682247

[B31] Liu C. Li Y. Semenov M. Han C. Baeg G. H. Tan Y. (2002). Control of Beta-Catenin Phosphorylation/degradation by a Dual-Kinase Mechanism. Cell 108 (6), 837–847. 10.1016/s0092-8674(02)00685-2 11955436

[B32] Liu A. Yu X. Liu S. (2013). Pluripotency Transcription Factors and Cancer Stem Cells: Small Genes Make a Big Difference. Chin. J. Cancer 32 (9), 483–487. 10.5732/cjc.012.10282 23419197 PMC3845564

[B33] Luo M. Shang L. Brooks M. D. Jiagge E. Zhu Y. Buschhaus J. M. (2018). Targeting Breast Cancer Stem Cell State Equilibrium through Modulation of Redox Signaling. Cell Metab. 28 (1), 69–e6. 10.1016/j.cmet.2018.06.006 29972798 PMC6037414

[B34] Lytle N. K. Ferguson L. P. Rajbhandari N. Gilroy K. Fox R. G. Deshpande A. (2019). A Multiscale Map of the Stem Cell State in Pancreatic Adenocarcinoma. Cell 177 (3), 572–e22. 10.1016/j.cell.2019.03.010 30955884 PMC6711371

[B35] Masuda M. Uno Y. Ohbayashi N. Ohata H. Mimata A. Kukimoto-Niino M. (2016). TNIK Inhibition Abrogates Colorectal Cancer Stemness. Nat. Commun. 7, 12586. 10.1038/ncomms12586 27562646 PMC5007443

[B36] Miller K. D. Nogueira L. Mariotto A. B. Rowland J. H. Yabroff K. R. Alfano C. M. (2019). Cancer Treatment and Survivorship Statistics, 2019. CA Cancer J. Clin. 69 (1), 363–385. 10.3322/caac.2155110.3322/caac.21565 31184787

[B37] Ordóñez-Morán P. Dafflon C. Imajo M. Nishida E. Huelsken J. (2015). HOXA5 Counteracts Stem Cell Traits by Inhibiting Wnt Signaling in Colorectal Cancer. Cancer Cell 28 (6), 815–829. 10.1016/j.ccell.2015.11.001 26678341

[B38] Park H. J. Park J. B. Lee S. J. Song M. (2017). Phellinus Linteus Grown on Germinated Brown Rice Increases Cetuximab Sensitivity of KRAS-Mutated Colon Cancer. Int. J. Mol. Sci. 18 (8), 1746. 10.3390/ijms18081746 PMC5578136 28800074

[B39] Patruno A. Tosco P. Borretto E. Franceschelli S. Amerio P. Pesce M. (2012). Thymopentin Down-Regulates Both Activity and Expression of iNOS in Blood Cells of Sézary Syndrome Patients. Nitric Oxide 27 (3), 143–149. 10.1016/j.niox.2012.06.002 22721692

[B40] Phi L. T. H. Sari I. N. Yang Y. G. Lee S. H. Jun N. Kim K. S. (2018). Cancer Stem Cells (CSCs) in Drug Resistance and Their Therapeutic Implications in Cancer Treatment. Stem Cell Int. 2018, 5416923. 10.1155/2018/5416923 PMC5850899 29681949

[B41] Polakis P. (2007). The many Ways of Wnt in Cancer. Curr. Opin. Genet. Dev. 17 (1), 45–51. 10.1016/j.gde.2006.12.007 17208432

[B42] Prieto-Vila M. Takahashi R. U. Usuba W. Kohama I. Ochiya T. (2017). Drug Resistance Driven by Cancer Stem Cells and Their Niche. Int. J. Mol. Sci. 18 (12), 2574. 10.3390/ijms18122574 PMC5751177 29194401

[B43] Rawla P. Sunkara T. Barsouk A. (2019). Epidemiology of Colorectal Cancer: Incidence, Mortality, Survival, and Risk Factors. Prz Gastroenterol. 14 (2), 89–103. 10.5114/pg.2018.81072 31616522 PMC6791134

[B44] Regan J. L. Schumacher D. Staudte S. Steffen A. Haybaeck J. Keilholz U. (2017). Non-Canonical Hedgehog Signaling Is a Positive Regulator of the WNT Pathway and Is Required for the Survival of Colon Cancer Stem Cells. Cell Rep. 21 (10), 2813–2828. 10.1016/j.celrep.2017.11.025 29212028

[B45] Roderick H. L. Cook S. J. (2008). Ca2+ Signalling Checkpoints in Cancer: Remodelling Ca2+ for Cancer Cell Proliferation and Survival. Nat. Rev. Cancer 8 (5), 361–375. 10.1038/nrc2374 18432251

[B46] Sabisz M. Skladanowski A. (2009). Cancer Stem Cells and Escape from Drug-Induced Premature Senescence in Human Lung Tumor Cells: Implications for Drug Resistance and *In Vitro* Drug Screening Models. Cell Cycle 8 (19), 3208–3217. 10.4161/cc.8.19.9758 19738435

[B47] Sahlberg S. H. Spiegelberg D. Glimelius B. Stenerlöw B. Nestor M. (2014). Evaluation of Cancer Stem Cell Markers CD133, CD44, CD24: Association with AKT Isoforms and Radiation Resistance in colon Cancer Cells. PLoS One 9 (4), e94621. 10.1371/journal.pone.0094621 24760019 PMC3997403

[B48] Singh V. K. Biswas S. Mathur K. B. Haq W. Garg S. K. Agarwal S. S. (1998). Thymopentin and Splenopentin as Immunomodulators. Current Status. Immunol. Res. 17 (3), 345–368. 10.1007/BF02786456 9638477

[B49] Terrié E. Coronas V. Constantin B. (2019). Role of the Calcium Toolkit in Cancer Stem Cells. Cell Calcium 80, 141–151. 10.1016/j.ceca.2019.05.001 31103948

[B50] Tirosh I. Venteicher A. S. Hebert C. Escalante L. E. Patel A. P. Yizhak K. (2016). Single-cell RNA-Seq Supports a Developmental Hierarchy in Human Oligodendroglioma. Nature 539 (7628), 309–313. 10.1038/nature20123 27806376 PMC5465819

[B51] Tominaga K. Minato H. Murayama T. Sasahara A. Nishimura T. Kiyokawa E. (2019). Semaphorin Signaling via MICAL3 Induces Symmetric Cell Division to Expand Breast Cancer Stem-like Cells. Proc. Natl. Acad. Sci. U S A. 116 (2), 625–630. 10.1073/pnas.1806851116 30587593 PMC6329980

[B52] Tosoni D. Pambianco S. Ekalle Soppo B. Zecchini S. Bertalot G. Pruneri G. (2017). Pre-clinical Validation of a Selective Anti-cancer Stem Cell Therapy for Numb-Deficient Human Breast Cancers. EMBO Mol. Med. 9 (5), 655–671. 10.15252/emmm.201606940 28298340 PMC5412856

[B53] Triana-Martínez F. Loza M. I. Domínguez E. (2020). Beyond Tumor Suppression: Senescence in Cancer Stemness and Tumor Dormancy. Cells 9 (2), 346. 10.3390/cells9020346 PMC7072600 32028565

[B54] Valkenburg K. C. Graveel C. R. Zylstra-Diegel C. R. Zhong Z. Williams B. O. (2011). Wnt/β-catenin Signaling in Normal and Cancer Stem Cells. Cancers (Basel) 3 (2), 2050–2079. 10.3390/cancers3022050 24212796 PMC3757404

[B55] Van der Jeught K. Xu H. C. Li Y. J. Lu X. B. Ji G. (2018). Drug Resistance and New Therapies in Colorectal Cancer. World J. Gastroenterol. 24 (34), 3834–3848. 10.3748/wjg.v24.i34.3834 30228778 PMC6141340

[B56] Wang X. Liu Q. Hou B. Zhang W. Yan M. Jia H. (2013). Concomitant Targeting of Multiple Key Transcription Factors Effectively Disrupts Cancer Stem Cells Enriched in Side Population of Human Pancreatic Cancer Cells. PLoS One 8 (9), e73942. 10.1371/journal.pone.0073942 24040121 PMC3770686

[B57] Wang J. Sun L. F. Cui W. W. Zhao W. S. Ma X. F. Li B. (2017). Intersubunit Physical Couplings Fostered by the Left Flipper Domain Facilitate Channel Opening of P2X4 Receptors. J. Biol. Chem. 292 (18), 7619–7635. 10.1074/jbc.M116.771121 28302727 PMC5418059

[B58] Wolf E. Milazzo S. Boehm K. Zwahlen M. Horneber M. (2011). Thymic Peptides for Treatment of Cancer Patients. Cochrane Database Syst. Rev. 2011 (2), CD003993. 10.1002/14651858.CD003993.pub3 PMC6481824 21328265

[B59] Xiaojing C. Yanfang L. Yanqing G. Fangfang C. (2017). Thymopentin Improves Cardiac Function in Older Patients with Chronic Heart Failure. Anatol J. Cardiol. 17 (1), 24–30. 10.14744/AnatolJCardiol.2016.6692 27564775 PMC5324858

[B60] Yang L. Shi P. Zhao G. Xu J. Peng W. Zhang J. (2020). Targeting Cancer Stem Cell Pathways for Cancer Therapy. Signal. Transduct Target. Ther. 5 (1), 8. 10.1038/s41392-020-0110-5 32296030 PMC7005297

[B61] Yang T. Wang P. Yin X. Zhang J. Huo M. Gao J. (2021). The Histone Deacetylase Inhibitor PCI-24781 Impairs Calcium Influx and Inhibits Proliferation and Metastasis in Breast Cancer. Theranostics 11 (5), 2058–2076. 10.7150/thno.48314 33500709 PMC7797697

[B62] Yu W. K. Wang Z. Fong C. C. Liu D. Yip T. C. Au S. K. (2017). Chemoresistant Lung Cancer Stem Cells Display High DNA Repair Capability to Remove Cisplatin-Induced DNA Damage. Br. J. Pharmacol. 174 (4), 302–313. 10.1111/bph.13690 27933604 PMC5289946

[B63] Zhang L. Wei X. Zhang R. Petitte J. N. Si D. Li Z. (2019). Design and Development of a Novel Peptide for Treating Intestinal Inflammation. Front. Immunol. 10, 1841. 10.3389/fimmu.2019.01841 31447849 PMC6691347

[B64] Zhao J. (2016). Cancer Stem Cells and Chemoresistance: The Smartest Survives the Raid. Pharmacol. Ther. 160, 145–158. 10.1016/j.pharmthera.2016.02.008 26899500 PMC4808328

[B65] Zhu L. Gibson P. Currle D. S. Tong Y. Richardson R. J. Bayazitov I. T. (2009). Prominin 1 marks Intestinal Stem Cells that Are Susceptible to Neoplastic Transformation. Nature 457 (7229), 603–607. 10.1038/nature07589 19092805 PMC2633030

